# Myosin VI and Associated Proteins Are Expressed in Human Macrophages but Do Not Play a Role in Foam Cell Formation in THP-1 Cells

**DOI:** 10.1155/2013/516015

**Published:** 2013-06-09

**Authors:** Hayley J. Dawson, Andrew P. Hibbert, Peter D. Chantler, Kathleen M. Botham

**Affiliations:** Department of Comparative Biomedical Sciences, Royal Veterinary College, Royal College Street, London NW1 0TU, UK

## Abstract

Myosin VI (Myo6) functions in endocytosis in conjunction with binding partners including adaptor protein (AP)-2, disabled 2 (Dab2), and GAIP interacting protein C terminus 1 (GIPC1). This study aimed to investigate the expression and function of Myo6 in macrophages and its possible role in the endocytosis of lipoproteins during the induction of foam cell formation. Expression of Myo6, AP-2 (**α**2 subunit), and Dab2 in THP-1 macrophages and primary human monocyte-derived macrophages was demonstrated at the mRNA and protein level, but GIPC1 was only detected at the mRNA level. Immunofluorescence showed that Myo6 was distributed similarly to F-actin in both macrophage types. AP-2**α**2 was found to have a similar subcellular distribution to Myo6 and Dab2 in THP-1 cells. Myo6 was located within membrane ruffles and protrusions of the plasma membrane. These results suggest that in macrophages Myo6 is required for several functions including cell adhesion, cell progression, and macropinocytosis. Low-density lipoprotein (LDL) and oxidised LDL (oxLDL) decreased Myo6 and GIPC1 mRNA expression in THP-1 cells, but uptake of the fluorescence-labelled lipoproteins was unaffected by knockdown of the expression of Myo6 or associated proteins with siRNA. Our findings, therefore, do not support the idea that Myo6 plays a major role in foam cell formation.

## 1. Introduction

The uptake of large amounts of lipids from plasma lipoproteins via endocytosis by macrophages in the artery wall to form lipid-engorged foam cells is an important initiating event in the development of atherosclerosis, a major cause of heart disease. The main lipoprotein implicated is low-density lipoprotein (LDL), but oxidative modification of the particles, a process which can occur within the vessel wall, is required before foam cell formation is induced [1].

The myosin superfamily is a group of actin-associated motor proteins that use ATP hydrolysis to generate force or directional movement along actin filaments [2, 3]. Many new classes of myosin have been identified since the discovery, over seventy years ago, of myosin II (“conventional” myosin), which produces contractile force in muscle cells; to date, 35 classes of myosin proteins have been characterised in mammalian cells [4]. It is now known that these various classes of myosin motor are involved in a diverse range of intracellular processes, ranging from cell migration and division to cell anchorage and transport of cargo proteins along actin filaments [3]. Myosin VI (Myo6), a member of the “unconventional” myosin classes, is an unusual member of the myosin superfamily in that its movement along actin filaments is towards the pointed (−) end of an actin filament, the opposite direction to myosins of other classes [5]. For this reason, Myo6 is ideally suited for a role in endocytosis, which involves vesicle formation and/or transport away from the plasma membrane [6]. Evidence suggests that it may play a part in organization and/or anchorage of endocytic machinery, in the provision of force required for invagination and/or vesicle fission, and during transport of vesicles through the cell cortex [6–8]. Attachment to vesicles is achieved through direct interaction with adaptor proteins (APs), including disabled 2 (Dab2) (which binds directly to receptors or indirectly via Adaptor Protein-2 (AP2)) and GAIP interacting protein C terminus 1 (GIPC1) [7–9].

Myo6 can be alternatively spliced in two regions of its cargo binding domain, giving rise to either a small insert (9aa) (SI) and/or a large insert (up to 32aa) (LI) [10]. Thus, four variants are possible: Myo6(+SI, +LI), Myo6(−SI, +LI), Myo6(+SI, −LI), and Myo6 without inserts (Myo6(−I)) [6]. These variants are differentially expressed and appear to have distinct cellular targets and functions [6, 7]. Although the inserts contain no functional motifs or binding sites, they may affect the structure and therefore the affinity of Myo6 for its binding partners [9, 11].

Endocytosis may occur by phagocytosis, the uptake of large particles, or by pinocytosis, the internalization of fluid and small particles [10, 12]. Phagocytosis is a regulated and selective process requiring receptor-mediated target recognition, but pinocytosis can be nonselective (macropinocytosis) or can be receptor-mediated. Clathrin-mediated endocytosis (CME) is the most studied form of receptor-mediated pinocytosis, but other forms include caveolin-mediated endocytosis, as well as clathrin-and-caveolin-independent endocytosis [10, 12]. Most evidence supporting a role for Myo6 in endocytosis relates to CME: it has been shown to colocalise with clathrin, Dab2, and AP-2 in polarised cells [13–15] and with uncoated vesicles and GIPC1 in unpolarised cells [13, 16]. In addition to CME, Myo6 has been found localized to sites of macropinocytosis in fibroblasts, and Holt et al. [17] have reported that loss of Myo6 function in bone marrow-derived murine dendritic cells causes enhancement of macropinocytotic uptake, but its exact function in this process remains unclear [7, 18].

Given the importance of endocytosis in the uptake of lipoproteins by macrophages, it is possible that Myo6 may have a role in foam cell formation and thus play a part in atherogenesis. Little is known, however, about the expression and function of Myo6 and its binding partners in macrophages. 

Expression of Myo6 [19], Dab2 [20], and AP-2 [21] has been detected in murine macrophages and expression of GIPC in human peripheral blood monocytes [22], but, except for one report which found Myo6 mRNA in macrophages derived from the human monocyte cell line, THP-1 [23], no information is available on expression of these proteins in human macrophages. Moreover, as far as we are aware, splice variant expression, subcellular localisation, and protein-protein interactions of Myo6 have not been studied previously in any macrophage type.

The aims of this study were to investigate the expression and function of Myo6 and related proteins in human macrophages and to test the hypothesis that Myo6 plays a role in the endocytosis of LDL and/or oxLDL by these cells. Expression of Myo6 (including splice variants), Dab2, AP-2*α*2 (one of the large subunits of the AP-2 heterotetrameric complex), and GIPC1 was demonstrated in both THP-1 macrophages and primary human monocyte-derived macrophages (HMDM), and their subcellular location and interactions within the cells were investigated. To study the possible role of these proteins in macrophage endocytosis, THP-1 macrophages were used to evaluate the effects of LDL and oxLDL on the expression of mRNA and protein for Myo6, Dab2, AP-2, and GIPC1, as well as the effects of inhibition of Myo6 and Dab2 expression by small interfering RNA (siRNA) on the uptake of the lipoproteins by the cells. The THP-1 cell line was established 30 years ago, and since then it has been used very extensively to study the role of monocyte/macrophages in cardiovascular disease. A recent comprehensive review of the value of this model [24] concluded that, in defined conditions, THP-1 cells resemble HMDM and mimic the changes seen in atherosclerosis and that, provided results are interpreted cautiously, they are suitable for the study of the mechanisms by which these cells affect vascular function. The present work shows that the expression and subcellular location of Myo6 and related proteins are generally similar in HMDM and THP-1 macrophages, but because of the practical difficulties in obtaining large numbers of HMDM, it was not possible to use primary cells in all of our experimental approaches.

## 2. Materials and Methods

### 2.1. Materials

Fetal bovine serum and penicillin/streptomycin were obtained from Gibco (Paisley, UK), and DMEM and L-glutamine were from PAA UK (Yeovil, UK). RPMI 1640 medium, *β*-mercaptoethanol, 4*β*-phorbol 12 myristate 13-acetate (PMA), Oil Red O, ethidium bromide, and fluorescein-isothiocyanate- (FITC-) conjugated goat anti-mouse IgG were supplied by Sigma Aldrich (Poole, UK). 4′,6-diamidino-2-phenylindole (DAPI) 1,1′-dioctadecyl-3,3,3′3′-tetramethylindo-carbocyanine perchlorate (DiI), rhodamine conjugated phalloidin, Alexa488 conjugated goat anti-rabbit-, and Alexa555 donkey anti-rabbit IgG were purchased from Invitrogen Molecular probes (Paisley, UK). Myo6 (H-215) and Dab2 (H-110) rabbit polyclonal IgG and normal donkey serum were from Santa Cruz Biotechnology Inc. (Heidelberg, Germany). Other antibodies including mouse polyclonal anti-AP-2*α*2 and rabbit polyclonal anti-*β*-microglobulin IgG (Abcam, Cambridge, UK), mouse polyclonal anti-GIPC1 IgG (Abnova, Heidelberg, Germany), rabbit polyclonal anti-*β*-actin (Cell Signaling Technology, Hitchin, UK), and horse radish peroxidase-conjugated goat anti-mouse and goat anti-rabbit IgG (Thermo Fisher Scientific, Cramlington, UK) were from various suppliers as indicated, and normal goat serum was from Dako UK Ltd (Ely, UK).

### 2.2. Culture of THP-1 Macrophages, HMDM and COS-7 Cells

THP-1 monocytes were cultured in suspension in RPMI-1640 media, supplemented with 10% heat inactivated (56°C, 30 min) fetal bovine serum (FBS), 1% penicillin/streptomycin, and 0.1%  *β*-mercaptoethanol (culture medium). For differentiation into macrophages, monocytes were incubated with 200 ng/mL phorbol 12-myristate 13-acetate (PMA) (200 ng/mL) for 72 h. Prior to experimental treatment, the medium containing PMA and nonadherent or dead cells was removed, the cells were washed twice, and fresh culture medium was added. Cells were incubated with or without treatment for up to 5 days, with media changes every 48 h.

For HMDM preparation, primary human monocytes were isolated from the blood of healthy adult volunteers with ethical approval from the East London Research Ethics Committee. Blood was collected into tubes containing 15% EDTA (v : v) and processed immediately by the addition of PBS (1 : 1, v : v), layering over 15 mL Lymphoprep (Axis Healthcare Ltd, Borehamwood, Herts, UK) and centrifugation at 800 ×g (30 min, 20°C). The mononuclear cell layer was collected, mixed with an equal volume of ice-cold PBS containing 0.4% (v/w) trisodium citrate, and centrifuged at 800 ×g (5 min, 4°C). After removal of the supernatant, the cell pellet was re-suspended in 0.2% (w : v) NaCl for 30 sec at 4°C to lyse any remaining red blood cells and 1.6% (w : v), NaCl was added, and the tubes were centrifuged as previously mentioned. Resuspension in PBS containing 0.4% (w : v) trisodium citrate and centrifugation were then repeated ×6 to remove contaminating platelets. The final pellet was resuspended in RPMI-1640 medium containing 5% FBS and 1% penicillin/streptomycin, and the cells were incubated at 37°C in 5% CO_2_ for 7 days to allow differentiation into macrophages. Examination by light microscopy indicated that the cells showed a macrophage phenotype after 6 days; cells were used on the 7th day after washing with PBS (×4) to remove any remaining nonadherent cells.

COS-7 adherent cells were cultured in DMEM maintenance media supplemented with 10% FBS, 1% penicillin/streptomycin, and 1% L-glutamine.

### 2.3. mRNA Analysis

Total RNA was extracted from HMDM, THP-1 macrophages, and COS-7 cells using an extraction kit (Sigma-Aldrich, Poole, UK) and RNase-free DNase (Qiagen, Crawley, UK) as recommended by the manufacturers. For reverse transcription, RNA (1 *μ*g) was used to generate cDNA using Omniscript Reverse transcriptase (Qiagen) and Oligo-(dT) primers according to the manufacturers' instructions. For expression studies in untreated cells, PCR was used to amplify gene products for Myo6, the small and large insert regions of Myo6, Dab2, AP-2*α*2, GIPC1, and in GeneAmp PCR System 9700 (Applied Biosystems) using the primers shown in Table 1 and the following protocol: denaturing at 95°C (5 min), followed by 35 cycles consisting of 30 sec at 94°C, 90 sec at the appropriate specific annealing temperatures and extension at 72°C for 60 sec, with a final extension at 72°C for 10 min. Product sizes and annealing temperatures are shown in Table 2. PCR products were visualised on 1.2% or 3% agarose gels containing 0.01% ethidium bromide (v : v). Gels were viewed and photographed under UV light using the ChemiDoc XRS scanner (Bio-Rad, Hemel Hempstead, UK).

To determine the effects of LDL or oxLDL on mRNA expression, THP-1 macrophages were incubated with the lipoproteins (50 *μ*g protein/mL) for 8 or 24 h, and total RNA was then extracted and reverse transcribed as described previously. The abundance of mRNA transcripts for Myo6, Dab2, AP-2*α*2, GIPC1, *β*-actin, *β*2-macroglobulin, and ribosomal protein L13a was assessed by quantitative PCR (qPCR) in an Opticon 2 LightCycler system (MJ Research, Waltham, Massachusetts, USA) using the primers shown in Table 1 employing the following protocol: denaturing at 94°C (2 min), followed by 37 cycles of amplification consisting of denaturation at 94°C (15 sec), incubation at an appropriate, specific annealing temperature (1 min), followed by extension at 72°C (1 min). Threshold cycle values were determined using Opticon Monitor 3 software and quantified using the standard curve for each gene. Normalisation factors (NF) were calculated from the geometric means of the three most stably expressed reference genes (*β*-actin, *β*2-macroglobulin, and ribosomal L13a protein) as determined using geNorm software or, for siRNA studies, to the reference gene *β*2-microglobulin run on the same qPCR plate with its own standard curve. In the latter case, annealing temperatures (Table 1) were adjusted to a temperature suitable for both the genes of interest and *β*2-microglobulin.

### 2.4. Western Blotting

For analysis of protein expression by Western blotting, cells were washed twice with PBS and treated with RIPA lysis buffer (150 mM NaCl, 50 mM Tris (PH 8.0), 1% Triton X-100, 0.5% deoxycholate (DOC), 0.1% SDS) containing protease inhibitor cocktail (10 *μ*L/mL) (5 min, 4°C). Lysates were agitated for 30 min at 4°C, centrifuged at 93 ×g (10 min at 4°C) to remove cell debris, and then denatured and reduced in NuPAGE LDS Sample Buffer (2 : 1, v : v) containing 10%  *β*-mercaptoethanol solution at 70°C for 10 min. Proteins (10–30 *μ*g) were separated by SDS-polyacrylamide electrophoresis (8% or 10% resolving gel with 5% stacking gel or precast Mini-PROTEAN TGX BioRad graded gels (4–15%) (BioRad)) and transferred to polyvinylidene fluoride (PDVF) membranes. Blocking was carried out in 5% (w : v) nonfat milk powder at room temperature for 1 h, and membranes were then incubated with polyclonal rabbit anti-Myo6 (1 : 200), anti-Dab2 (1 : 400), anti-*β*-actin (1 : 1,000) or anti-*β*-microglobulin (1 : 700), or mouse anti-GIPC1 (1 : 500) IgG overnight at 4°C with agitation. After washing in TBST (4 × 15 min), horseradish peroxidase-conjugated goat anti-rabbit or anti-mouse secondary antibodies (*β*2-microglobulin, Dab2, Myo6, 1 : 8,000; *β*-actin, GIPC1, 1 : 10,000) were added and incubations were continued for 1 h at room temperature with agitation. Finally membranes were washed in TBST (4 × 15 min) and bands were visualized using enhanced chemiluminescence (GE healthcare, Little Chalfont, UK). Band densities were digitized from film to 12 bit images using a ChemiDoc XRS (Bio-Rad) and quantified as mean grey values measured over identical areas using Quantity One software (Bio-Rad). Results are shown relative to the control sample in each case.

### 2.5. Confocal Laser Scanning (CLS) Fluorescence Microscopy

CLS fluorescence microscopy was conducted using either a Leica SP5 or a Zeiss LSM510 confocal microscope. For all experiments, THP-macrophages or HMDM were incubated in 24-well plates containing coverslips, which were removed for mounting at the end of the procedure. Coverslips were washed twice in 1 × PBS, fixed with 4% formalin (v : v) for 15 min, and followed by cell permeabilisation in 0.25% Triton X-100 for 5 min prior to blocking (1 h, 3% (w : v) BSA) with normal goat (NGS) or donkey (NDS) serum (1% w : v). IgG polyclonal rabbit anti-Myo6, -GIPC1 (1 : 100, v : v), -Dab2 (1 : 200, v : v), or polyclonal mouse anti-AP-2*α*2 (1 : 100, v : v) (diluted in 0.1% (w : v) BSA and 1% NGS/NDS in PBS) was then added. After 1 h, cells were washed (0.5% BSA in PBS, 5 min × 4 and 15 min × 1) and incubated with IgG polyclonal secondary antibodies conjugated to a specific fluorochrome as follows: goat anti-rabbit antibody conjugated to Alexa Fluor 488 (1 : 1,000, v : v), donkey anti-rabbit antibody conjugated to Alexa Fluor 555 (1 : 1,000, v : v), or goat anti-mouse antibody conjugated to Fluorescein Isothiocyanate (FITC) (1 : 25, v : v) dilutions in 0.1% BSA and 1% NGS in PBS (w : v). Where double immunofluorescence staining was used, compatible pairs of primary or secondary antibodies were incubated with cells simultaneously. Cells were then washed in PBS (5 min × 3, then 15 min × 1).

To visualise cell nuclei, cells were incubated (3 min) with 300 nM 4′,6-diamidino-2-phenylindole (DAPI), a nucleic acid stain. The structure of F-actin was preserved prior to visualisation, with minimal loss of free myosin, by incubating cells (45 sec) with buffer containing NaCl, 137 mM; KCI, 5 mM; Na_2_HPO_4_, 1.1 mM; KH_2_PO_4_, 0.4 mM; NaHCO_3_, 4 mM; glucose, 5.5 mM; MgCl_2_, 2 mM; EGTA, 2 mM; PIPES, 5 mM; pH 6.0-6.1 [25, 26] supplemented with 0.32 M sucrose, 0.1% Triton X-100, and 1 *μ*g/mL phalloidin [27]. Cells were immediately incubated with 165 nM phalloidin conjugated to rhodamine, as a fluorescent marker (20 min). Phalloidin binding to actin stabilises the filament, preventing depolymerisation. To remove residual counterstain, cells were washed twice in PBS before mounting. Areas of fluorescence signal were detected by application of a threshold and the mean fluorescence intensity quantified using Volocity (5.4) 3D image analysis software (PerkinElmer, Waltham, MA, USA).

### 2.6. Low-Density Lipoprotein (LDL) Isolation and Modification

LDL (*d* = 1.019–1.063 g/mL) was isolated from human plasma obtained from the National Blood Service (North London, UK), using sequential density gradient ultracentrifugation. Final preparations of native LDL (nLDL) were dialysed against 0.9% NaCL (w/v) containing 10 *μ*M EDTA (5L × 4). For LDL used in oxidation studies, EDTA was omitted. Lipoproteins were stored at 4°C, protected from light and under argon gas. Human plasma was used within 1 month of purchase and LDL within 2 weeks of isolation. 

LDL was oxidised by incubation with CuSO_4_ (5 *μ*M) for 6 h, oxidation was terminated by the addition of EDTA (1%, 50 *μ*L/mL v/v), and the extent of oxidation was assessed by measuring the concentration of malondialdehyde (MDA) (nmol/mg LDL protein) using the Thiobarbituric Acid Reactive Substances (TBARS) assay [28]. CuSO_4_ was removed by dialysis at 4°C against 0.9% NaCl (w/v) containing 10 *μ*M EDTA (5L × 4). Both nLDL and oxLDL were sterilised by passage through a 0.45 *μ*m filter.

For labelling of nLDL and oxLDL with the fluorescent probe 1,1′-dioctadecyl-3,3,3′,3′-tetramethylindo-carbocyanine perchlorate (DiI), lipoprotein deficient serum (LPDS), prepared from human plasma by sequential density gradient ultracentrifugation, was added to the LDL preparations (1 mg LDL protein/2 mL LPDS) followed by DiI (300 *μ*g DiI/mg LDL protein). To prevent oxidation, EDTA was added to the preparations at a final concentration of 10*μ*M, and the mixture was incubated for 8 h at 37°C with shaking. The DiI labelled lipoproteins (DiI-nLDL/oxLDL) were then isolated by ultracentrifugation at *d* 1.063 g/mL (96,919 ×g, 15 h, 4°C) and dialysed as described previously before use.

### 2.7. siRNA Studies

Small interfering RNA (siRNA) sequences designed to target Myo6 (combination of two sequences), Dab2, AP-2*α*2 (Table 2), or GIPC1 (sequence not released by the manufacturer) were used to inhibit the expression of their cognate genes. THP-1 macrophages (6 × 10^5^ well) were transfected with siRNA using HiPerFect transfection reagent (Qiagen, Crawley, UK). 5 nM (AP-2*α*2, GIPC1, Dab2) or 40 nM (Myo6) siRNA was added to 100 *μ*L RIPA-1640 medium, then 20 *μ*L HiPerFect was added, and the mixture was incubated for 10 min at room temperature to allow siRNA-HiPerfect complexes to form prior to their addition (100 *μ*L) to cells. In all experiments, a nonsilencing scrambled siRNA (AllStars negative control, Qiagen), which has no known homology to any mammalian genes, was used as a control. To assess the effects of inhibition of gene expression on the uptake of nLDL and oxLDL by THP-1 macrophages, lipoproteins were labelled with the fluorescent probe DiI and uptake was assessed using fluorescence wide-field microscopy. Cells were seeded in 24-well plates (1.6 × 10^5^ cells/well) and incubated with siRNA for 48–72 h. DiI-labelled LDL or oxLDL (50 *μ*g protein/mL) was then added, and the incubations, continued for periods up to 24 h. Cells were fixed and imaged using a Leica inverted DMRIB wide-field microscope.

### 2.8. Analytical Methods

Protein concentrations of cell lysates were determined by the method of Lowry [29] and those of nLDL and oxLDL were obtained using Peterson's modification of Lowry's method [30]. The total cholesterol content of lipoproteins was determined by enzymatic analyses using commercially available kits (Thermo Fisher Scientific, Cramlington, UK). Statistical analysis was performed using Student's *t* test or one- or two-way ANOVA (followed by Bonferroni's multiple comparison test), as indicated in the text.

## 3. Results

### 3.1. Expression of Myo6 and Associated Proteins AP2, Dab2, and GIPC1 in THP-1 Macrophages and HMDM

**Figure 1:**
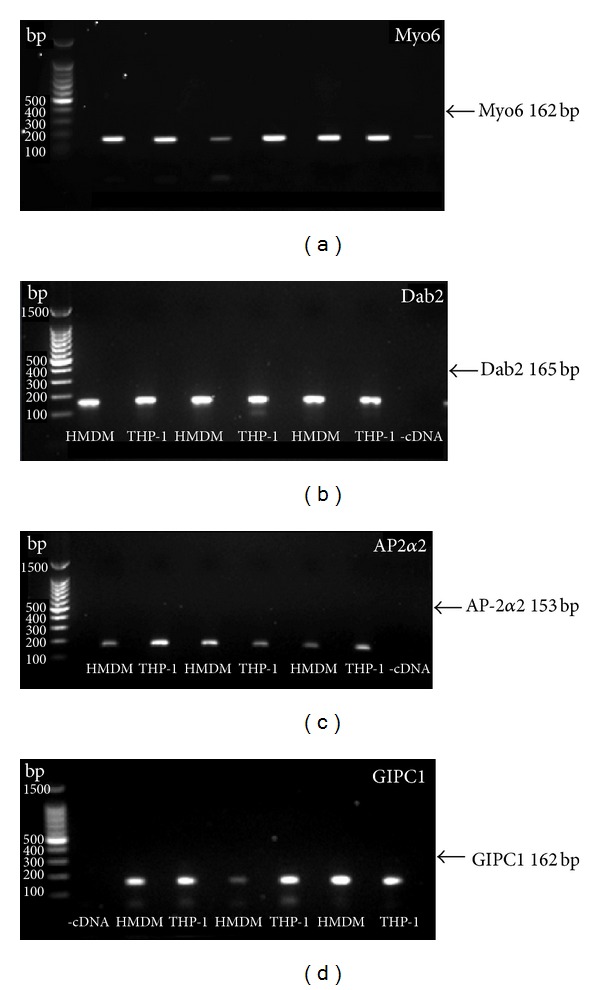
Expression of mRNA for Myo6 and related proteins in THP-1 macrophages and HMDM. Total RNA was extracted from THP-1 macrophages (3 separate lysates) and HMDM (obtained from three individual donors). PCR was used to amplify gene products for Myo6 (a), Dab2 (b), AP-2*α*2 (c), and GIPC1 (d). Products were visualised on a 1.2% agarose gel containing 0.01% ethidium bromide (v : v).

To determine whether Myo6 and its binding partners are expressed in macrophages, total RNA was isolated from untreated THP-1 macrophages and HMDM and then used to generate cDNA for amplification of gene products by conventional PCR. Bands at the expected product size (Table 1) were detected for Myo6 (Figure 1(a)) and also for Dab2 (Figure 1(b)), the large subunit of AP-2, AP-2*α*2, which was used to detect AP-2 expression (Figure 1(c)) and GIPC1 (Figure 1(d)). In all cases, no bands were found for any of the genes when PCR was carried out in the absence of either primers (data not shown) or cDNA. These results demonstrate that both THP-1 macrophages and HMDM express mRNA encoding Myo6, Dab2, AP-2*α*2, and GIPC1.

**Figure 2:**
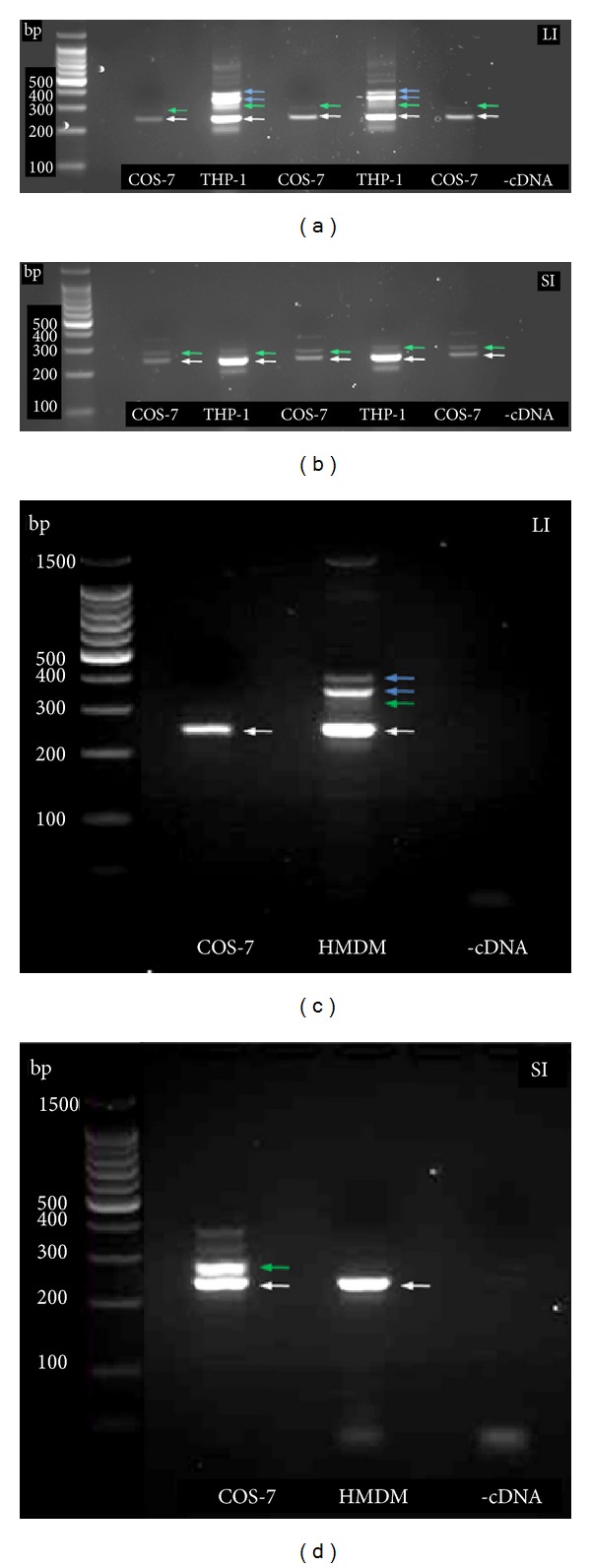
Expression of Myo6 splice variants in THP-1 macrophages and HMDM Total RNA was extracted from THP-1 macrophages (2 separate lysates), HMDM (from two individual donors), and COS-7 cells (3 separate lysates), and PCR was used to amplify the LI and SI of the Myo6 C-terminal tail domain. Products were visualised on a 1.2% agarose gel containing 0.01% ethidium bromide (v : v). (a) Myo6 LI in THP-1 macrophages and COS-7 cells; (b) Myo6 SI in THP-1 macrophages and COS-7 cells; (c) Myo6 LI in HMDM (1 of 2 experiments shown) and COS-7 cells; (d) Myo6 SI in HMDM (1 of 2 shown) and COS-7 cells. White arrows indicate Myo6(−LI), green arrows Myo6(+LI) (a, c) or Myo6(+SI) (b, d), and blue arrows alternatively spliced variants of Myo6 LI.

Although previous studies in mammalian cells have established that Myo6 can be expressed as four variants due to alternative splicing of the LI and SI regions of the tail domain [6, 11], it is not known which splice variants are expressed in macrophages. Dance et al. [11] demonstrated the presence of Myo6 both with and without the LI and SI in COS-7 cells, and we have used the same primer sequences to investigate their expression in THP-1 macrophages and HMDM. We found bands identical to those reported by Dance et al. [11] for Myo6 variants in COS-7 cells (Figure 2), and these were used as markers of band identity. Figure 2 shows that bands corresponding to Myo6(−LI) (Figure 2(a)) and Myo6(−SI) (Figure 2(b)) in COS-7 cells were also found in THP-1 macrophages (Figures 2(a) and 2(b), white arrows). A band of product size equivalent to Myo6(+SI) mRNA (Figure 2(b), green arrows) was also detected in THP-1 cells, although at a lower level relative to Myo6(−SI) in comparison to COS-7 cells. Expression of Myo6(+LI) was low in COS-7 cells, but a band corresponding to this variant was clearly present in THP-1 macrophages, and these cells also expressed number of other transcripts of larger size (Figure 2(a), blue arrows). HMDM showed similar patterns of expression of Myo6 splice variants to those observed in THP-1 cells, with Myo6(−LI) and multiple bands of Myo6(+LI) (Figure 2(c)) and Myo6(−SI) (Figure 2(d)) present; however, Myo6(+SI) was not detected in these cells (Figure 2(d)).

**Figure 3:**
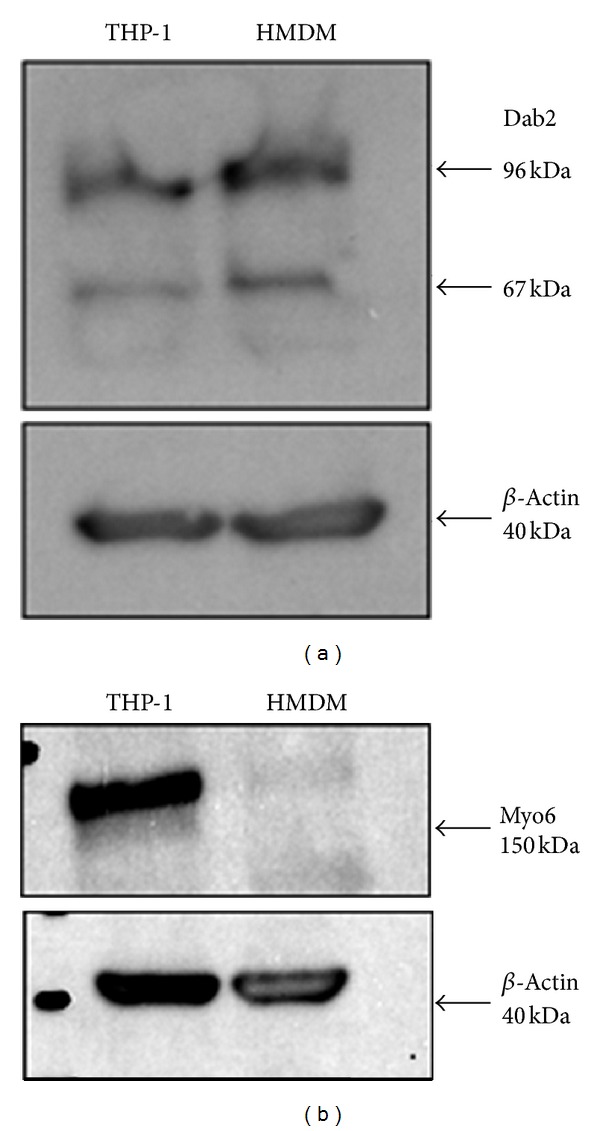
Expression of protein for Dab2 and Myo6 in THP-1 macrophages and HMDM. THP-1 macrophages or HMDM were lysed with RIPA buffer containing protease inhibitors. Protein expression was assessed by immunoblotting. (a) Dab2 (45 *μ*g protein), (b) Myo6 (45 mg protein) (Myo6 (H-215) and Dab2 (H-110) rabbit polyclonal IgG were from Santa Cruz Biotechnology Inc.). Results shown are representative of 3 separate experiments with THP-1 macrophages or cells from 2 individual donors for HMDM.

Immunoblotting indicated the presence of a protein of the molecular weight of Dab2 (96/67 kDa) in 3 lysates from THP-1 macrophages and in cells from 3 individual donors for HMDM. Images from a representative experiment are shown in Figure 3(a). In addition, a band corresponding to the molecular weight of Myo6 was clearly present in 3 lysates of THP-1 cells (one example is shown in Figure 3(b)). For HMDM, a weak band corresponding to Myo6 was visible in cells from 2 donors when 45 *μ*g protein was loaded (one example is shown in Figure 3(b)) but not when a lower amount of protein (35 *μ*g) was used (data not shown). The expression of Myo6 in HMDM, however, was clear in the immunofluorescence experiments described later.

We were unable to demonstrate the presence of GIPC1 protein definitively. In addition, AP-2*α*2 protein could not be detected by immunoblotting, although its presence in THP-1 macrophages was demonstrated by immunofluorescence (see later).

### 3.2. Subcellular Localisation of Myo6 and Associated Proteins in THP-1 Macrophages and HMDM

Immunofluorescence was used to explore the distribution and interactions of Myo6 and associated proteins in THP-1 macrophages and HMDM. The proteins were detected using Alexa488- (Myo6, Dab2) or FITC-(AP-2*α*2) conjugated secondary antibodies (green) and where appropriate, DAPI (blue) and rhodamine-conjugated phalloidin (red) were employed as counterstains for the nucleus and F-actin, respectively. No green fluorescence was observed in negative controls when cells were incubated with FITC- or Alexa488-conjugated secondary Ab in the absence of the primary antibodies.

**Figure 4:**
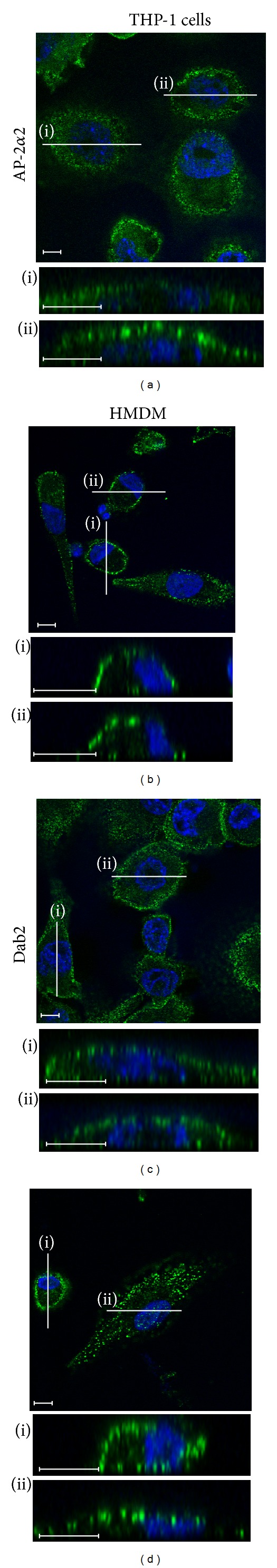
Subcellular location of AP-2, Dab2, and Myo6 in THP-1 macrophages and HMDM. THP-1 macrophages (a, c) or HMDM (b, d) were fixed and probed with IgG polyclonal mouse anti-AP-2*α*2 followed by FITC-conjugated anti-mouse antibody (green) (a, b) or IgG polyclonal rabbit anti-Dab2 followed by anti-rabbit antibody conjugated to Alexa488 (green) (c, d); all cells were counterstained with DAPI (blue) to highlight cell nuclei. Z-stacked images were taken using a Leica SP5 CLS microscope. The main panels show a single xy section through the cells. Below each, xz or yz vertical sections (i and ii) through individual cells are shown. Lines on the main panels show from where these cross-sections are taken. Typical images taken from a minimum of three separate experiments with THP-1 cells, or HMDM from three individual donors, are shown. Scale bars = 10 *μ*m.

**Figure 5:**
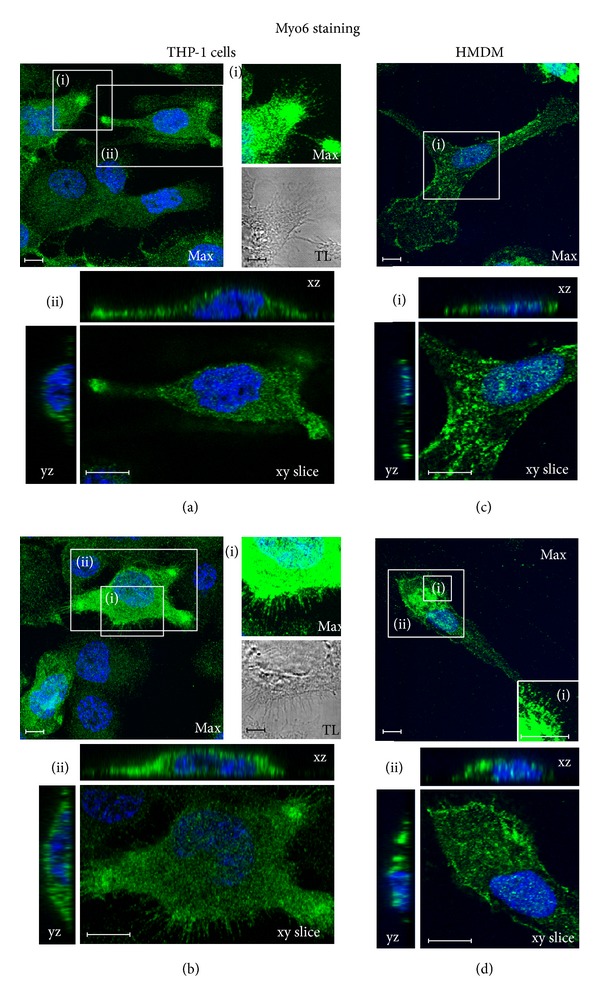
THP-1 macrophages (a, b) or HMDM (c, d) were fixed and probed with IgG polyclonal rabbit anti-Myo6 followed by anti-rabbit antibody conjugated to Alexa488 (green) and counterstained with DAPI (blue) to highlight cell nuclei. Z-stacked images were taken using a Leica SP5 CLS microscope. (a)-(b) THP-1 cells, labeled as described previously, are shown in maximum projections through the stacks, provided for orientation. Enlargements (i) of the regions indicated show cellular projections labeled for Myo-6. These projections are also visible in the transmitted light images (TL). Selected cells (ii) are enlarged below the maximum projections and shown as confocal xy slices with vertical xz and yz sections through the middle of the cells. (c)-(d) HMDM cells labeled as described previously are illustrated in maximum projections through the stacks. Selected regions (c (i), d (ii)) are enlarged and shown as confocal xy slices with vertical xz and yz sections through the middle of the cells. One region (d (i)) is enlarged as an inset to illustrate staining in short cellular projections. Typical images taken from a minimum of three separate experiments with THP-1 cells, or HMDM from three individual donors, are shown. Scale bars = 10 *μ*m.

AP-2*α*2 and Dab2 were located mainly around the cell periphery in a punctate staining pattern in both THP-1 macrophages (Figures 4(a) and 4(c)) and HMDM (Figures 4(b) and 4(d)). By contrast, Myo6 was found to exhibit a more diffuse staining in both cell types (Figure 5) and was found within membrane protrusions and membrane ruffles along the leading edge of the cells (THP-1 cells Figures 5(a), and 5(b), HMDM, Figures 5(c) and 5(d)).

**Figure 6:**
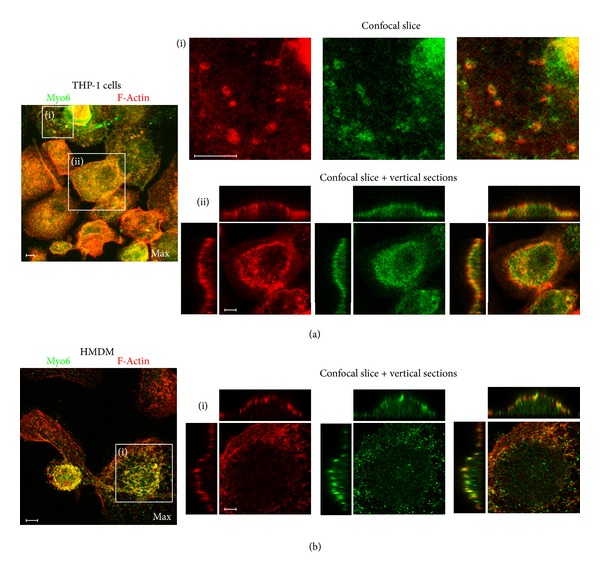
Relative distributions of Myo6 and F-actin in THP-1 macrophages and HMDM. THP-1 macrophages (a) or HMDM (b) were fixed and probed using double immunofluorescence for Myo6 (secondary antibody conjugated to Alexa488 (green)) and F-actin (visualised using rhodamine-conjugated phalloidin (red)); Z-stacked images were taken on a Zeiss LSM or Leica SP5 confocal microscope. Maximum projections are shown on the left for orientation. (a) (i) shows a confocal slice at the level of the bottom of the cells, taken from the region indicated to the left. The individual channels for F-actin (red), Myo6 (green), and the overlay are shown side-by-side. (a) (ii) and (b) (i) show single confocal sections and vertical xz and yz sections through the centre of the cells indicated to the left. The individual channels for F-actin (red), Myo6 (green), and the overlay are shown side-by-side. Typical images from at least 3 separate experiments with of THP-1 cells or HMDM from 3 individual donors are shown. Scale bars = 10 *μ*m.

**Figure 7:**
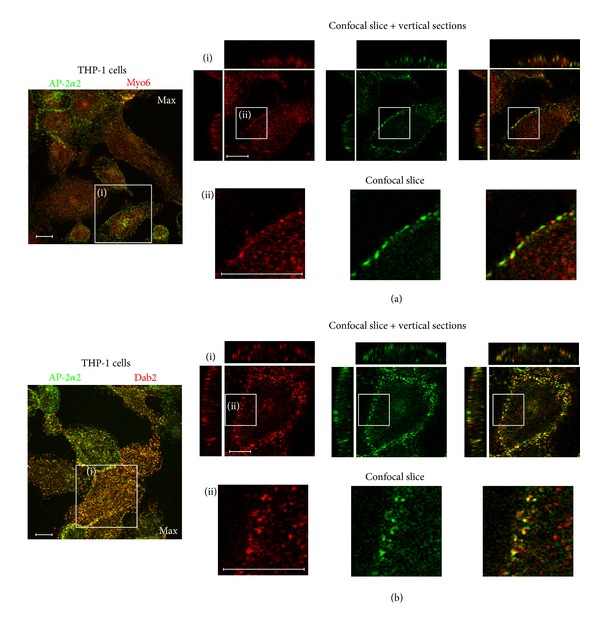
Relative distributions of AP-2 and Myo6 or Dab2 in THP-1 macrophages. THP-1 macrophages were fixed and probed using double immunofluorescence for (a) AP-2*α*2 (secondary antibody conjugated to FITC (green)) and Myo6 (secondary antibody conjugated to Alexa555 (red)); (b) AP-2*α*2 (secondary antibody conjugated to FITC (green)) and Dab2 (secondary antibody conjugated to Alexa555 (red)). Z-stacked images were taken on a Leica SP5 confocal microscope. Maximum projections are shown on the left for orientation. Series labeled (i) show single confocal sections and vertical xz and yz sections through the centre of the cells indicated to the left. The individual channels for Myo-6 or Dab2 (red), AP-2*α*2 (green), and the overlay are shown side-by-side. Enlargements of the single confocal slice, showing the plasma membrane, are shown in series (ii) and show the regions indicated in (i). Typical images from at least 3 separate experiments with THP-1 cells or HMDM from 3 individual donors are shown. Scale bars = 10 *μ*m.

Association between Myo6 and F-actin was studied using fluorescence labelling techniques in both THP-1 macrophages and HMDM (Figure 6). Confocal images showed that, as expected, Myo6 appeared to be associated with F-actin, with similar distributions mainly found around the cell periphery in both types of macrophages (Figures 6(a)(ii) and 6(b)), including possible podosome attachment sites in THP-1 cells (Figure 6(a)(i)). To assess the extent of interaction between Myo6 and AP-2*α*2, double immunofluorescence experiments were carried out in THP-1 macrophages in which Myo6 was labelled with Alexa555-linked secondary antibody to give red fluorescence (Figure 7(a)). AP-2*α*2 immunofluorescence was observed mainly at the cell periphery, while that for Myo6 was also found intracellularly (Figure 6(a)). Regions of overlap were rare and imperfect (Figure 7(a)(ii)), suggesting that the two proteins are not substantially associated within the cells. Experiments using an Alexa555-linked secondary antibody for Dab2, however, indicated that there was substantial colocalisation between AP-2*α*2 and Dab2 (Figure 7(b)).

### 3.3. Effects of LDL and oxLDL on the Expression of Myo6 and Related Proteins in THP-1 Macrophages

The compositions (protein, total cholesterol, and TBARS content) of the nLDL and oxLDL preparations used are shown in Table 3. No significant differences were found in the protein or total cholesterol content of nLDL as compared to oxLDL. The concentration of TBARS, however, was approximately 19-fold higher in oxLDL, indicating a significantly increased oxidative state.

**Figure 8:**
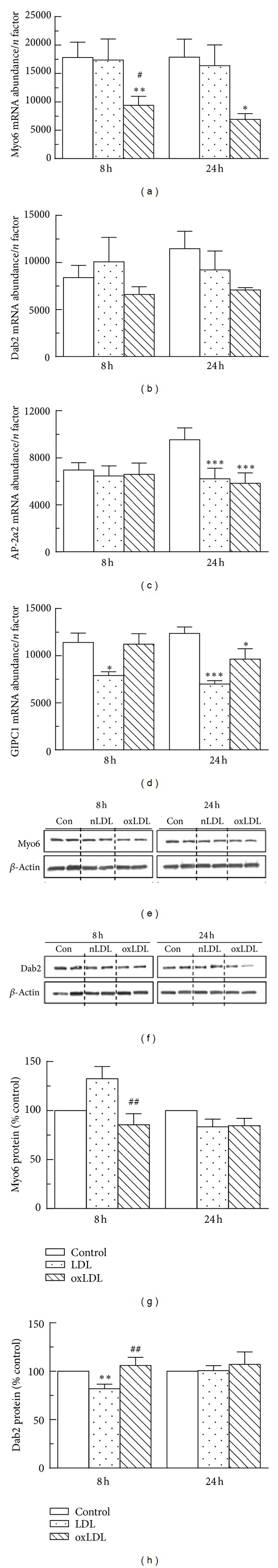
Effects of LDL and oxLDL on expression of mRNA and protein for Myo6 and related proteins in THP-1 macrophages. THP-1 cells were incubated with nLDL or oxLDL (50 *μ*g protein/mL) or an equal volume of PBS (control) for 8 h or 24 h, and total RNA or protein was isolated. The abundance of transcripts for (a) Myo6, (b) Dab2, (c) AP-2*α*2, and (d) GIPC1 was determined by qPCR (quantified using a standard curve and normalised using geNorm), and the expression of protein for (e) Myo6 and (f) Dab2 was assessed by immunoblotting using *β*-actin as the housekeeping gene (Con, control). Band density was quantified using Quantity One software (Biorad), and results for (g) Myo6 and (h) Dab2 are expressed as % control value. Data shown ((a)–(d), g, h) are the mean from 4 separate experiments and error bars show the SEM. Significance limits *P < 0.05, **P < 0.01, ***P < 0.001 versus control; ^#^ *P* < 0.05, ^##^ *P* < 0.01 versus nLDL (one-way ANOVA).

THP-1 macrophages were incubated with or without nLDL or oxLDL (50 *μ*g protein/mL) for 8 h or 24 h, and lysates were then used to determine the abundance of transcripts for Myo6, Dab2, AP-2*α*2, and GIPC1 by a combination of RT-qPCR and immunoblotting (the latter for Myo6 and Dab2). Incubation with nLDL caused no significant change in Myo6 or Dab2 mRNA levels at either time point (Figures 8(a) and 8(b)). mRNA abundance for AP-2*α*2, however, was decreased by about 35% after 24 h (*P* < 0.001) and that for GIPC1 by 31% after 8 h (*P* < 0.05) or 39% after 24 h (*P* < 0.001) (Figures 8(c) and 8(d)). oxLDL, however, caused a marked decrease in mRNA levels for Myo6 at both time points (8 h, −47%, *P* < 0.01; 24 h, −61%, *P* < 0.05) and also significantly reduced mRNA levels for AP-2*α*2 (−39%, *P* < 0.001) and GIPC1 (−22%, *P* < 0.05) compared to control values.

Myo6 and Dab2 protein concentrations showed little change when macrophages were incubated with either nLDL or oxLDL, with a small decrease in Dab2 (−19%) in the presence of nLDL being the only difference from control values (Figures 8(e)–8(h)). Myo6 proteins levels, however, were significantly lower after treatment with oxLDL as compared to nLDL after 8 h (+47%, *P* < 0.01, Figure 8(f)), while Dab2 levels were significantly higher (+24%, *P* < 0.01, Figure 8(h)).

### 3.4. siRNA Studies

**Figure 9:**
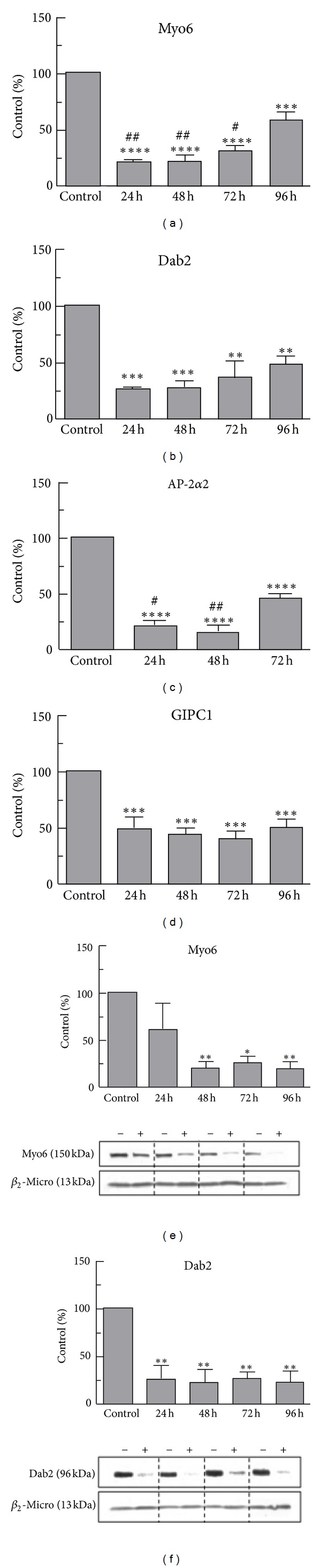
Inhibition of the expression of mRNA and protein for Myo6 and related proteins by siRNA. THP-1 macrophages were transfected with siRNA targeting Myo6, Dab2, AP-2*α*2, GIPC1 or a nonsilencing scrambled siRNA sequence (control) using HiPerFect transfection reagent. Cells were lysed at 24 h–96 h, and mRNA transcript levels for (a) Myo6, (b) Dab2, (c) AP-2*α*2, and (d) GIPC were determined by qPCR, and the expression of protein for (e) Myo6 and (f) Dab2 was assessed by immunoblotting. Band density was quantified using Quantity One software (Biorad), and results are expressed as % control value. +, siRNA; − scrambled siRNA. Data shown are the mean from 3 separate experiments, and error bars show the SEM. Significance limits, *P < 0.05, **P < 0.01, ***P < 0.001, ****P < 0.0001 versus control; ^#^ *P* < 0.05, ^##^ *P* < 0.01 versus 96 h (one-way ANOVA).

The effects of inhibition of the expression of Myo6, Dab2, AP-2*α*2, and GIPC1 on the uptake of nLDL and oxLDL by THP-1 macrophages were studied using siRNA sequences designed to target their cognate genes. mRNA abundance for all test proteins was maximally decreased in THP-1 macrophages 24–48 h after transfection with antisense oligonucleotides as compared to scrambled controls (reduction in abundance was −75–85%, Myo6, Dab2, and AP-2*α*2, *P* < 0.0001; −50% GIPC, *P* < 0.001) (Figures 9(a)–9(d)). After 72 h (AP-2*α*2) or 96 h (Myo6 and Dab2), however, the inhibitory effect was reduced for all genes except GIPC1. In addition, protein concentrations of Myo6 were reduced by 75–83% between 48 and 96 h and those for Dab2 by >75% between 24 and 96 h (*P* < 0.01) (Figures 9(e) and 9(f)). 

**Figure 10:**
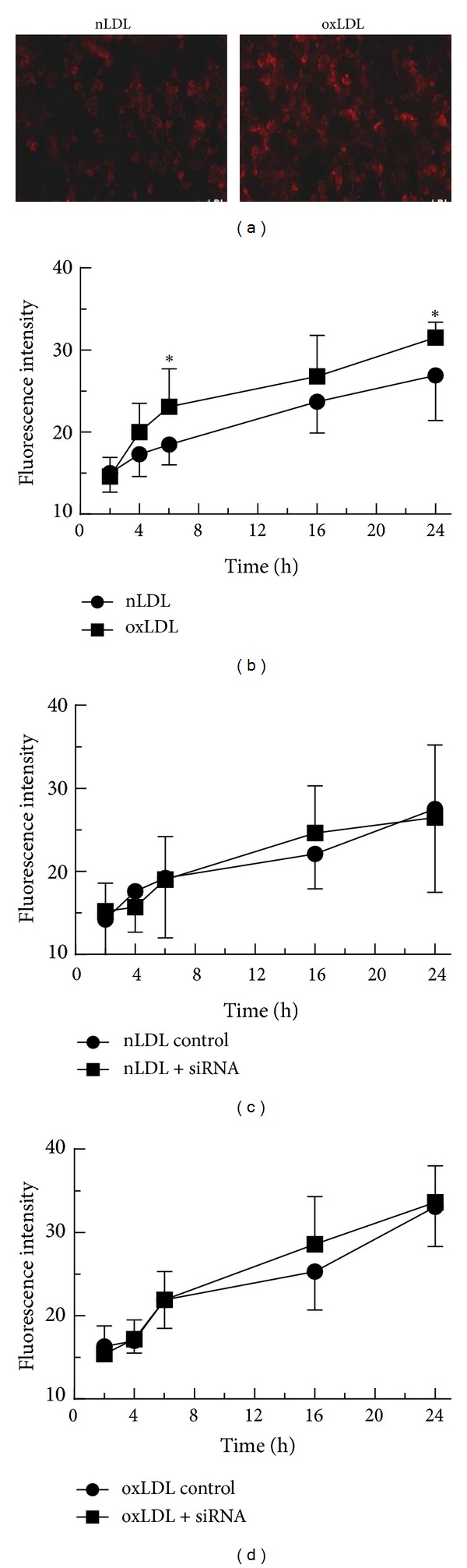
Effect of siRNA targeted to Myo6 on the uptake of LDL and oxLDL by THP-1 macrophages. (a), (b) THP-1 macrophages were incubated with nLDL or oxLDL (50 *μ*g protein/mL) for 6 h (a) or time points up to 24 h (b), and the area of fluorescent signal associated with the cells was assessed using a Leica inverted DMIRB wide-field fluorescence microscope. (c), (d) THP-1 cells were transfected with siRNA targeting Myo6 or with a nonsilencing scrambled siRNA sequence (control) using HiPerFect transfection reagent. After 48 h, cells were treated with 50 *μ*g protein/mL DiI-labelled nLDL (c) or oxLDL (d) and fixed at time points up to 24 h. Wide-field images were taken using a Leica DMRIB fluorescence microscope. The mean intensities of the fluorescent signals ((b)–(d)) were measured using Volocity software. Data shown are the means from three separate experiments, and error bars show the SEM. Significance limits, *P < 0.05 versus nLDL (two way ANOVA).

Uptake of nLDL and oxLDL by THP-1 macrophages was measured using DiI-labelled lipoproteins (50 *μ*g/mL) and fluorescence microscopy. In non-siRNA-treated cells, the area of fluorescence signal increased between 2 h and 24 h, and, as expected, the rate of increase was faster with oxLDL as compared to nLDL (Figures 10(a) and 10(b)). Comparison of the areas of fluorescence signal in macrophages transfected with scrambled siRNA, or siRNA targeting Myo6 showed no significant differences (Figures 10(c) and 10(d)); similar results were obtained when siRNA targeting Dab2, AP-2*α*2 or GIPC1 were used (data not shown).

## 4. Discussion

Myo6 is an intracellular motor protein found to be associated with F-actin in the cytoskeleton [7, 31]; in cells where it functions in endocytosis, it is also found in association with other proteins that have assigned roles in endocytosis, namely, AP-2, Dab2, and/or GIPC1. Myo6 and the interactive adaptor proteins Dab2, AP-2, and GIPC1 are widely expressed and play diverse, often essential, roles in cellular functioning and signalling. Furthermore, these proteins have been shown to function and interact during CME, and Myo6 is thought to provide a driving force for vesicle formation and trafficking [6]. However, although the endocytosis of lipoproteins by macrophages to form foam cells is crucial to atherosclerotic development, the potential roles for Myo6 and binding partners in this process have not been explored till now. Indeed, little information is available about the expression of these proteins or their roles in human macrophages, and nothing is known about their subcellular location or mutual interactions in these cells.

We have demonstrated the mRNA expression for Myo6, Dab2, AP-2, and GIPC1 in primary human macrophages (HMDM) as well as in macrophages derived from the human monocyte cell line, THP-1 (Figure 1). Furthermore, the presence of Myo6, Dab2, and AP-2 protein was demonstrated in these two cell types using both immunoblotting and immunofluorescence (Figures 3–5). Previous studies have detected Dab2 mRNA in mouse bone marrow macrophages and Myo6, Dab2, and AP-2 protein in various murine macrophage cell lines [19–21, 32]. In addition, Myo6 mRNA [33] and low levels of GIPC1 mRNA [34] or protein [22] have been found in human peripheral blood leukocytes. Nonetheless, this is the first report of the expression of these proteins in human macrophages, except for one study showing Myo6 mRNA expression in THP-1 cells [23]. 

As positive controls, we demonstrated splice variant expression of Myo6 in COS-7 cells, previously shown to express Myo6 with and without the LI and SI insert sequences [11]. THP-1 macrophages and HMDM were found to express Myo6(−LI), but while only one weak band corresponding to Myo6(+LI) was observed in COS-7 cells, numerous bands in this region were seen within the two macrophage cell lines (Figures 2(a) and 2(c)). Dance et al. [11] found a similar pattern of multiple bands for Myo6(+LI) in the epithelial cell lines ARPE-19 and LLC-PK_1_ and determined that these resulted from alternative splicing within the LI region. Expression of Myo6(−SI) was demonstrated in THP-1 macrophages and HMDM in our experiments (Figures 2(b) and 2(d)), but although Myo6(+SI) was clearly present in THP-1 cells, its expression was not detected in HMDM. Alternative splice variants of Myo6 are differentially expressed and have distinct subcellular locations and functions in various cell types. In polarised epithelial cells, for example, Myo6(−LI) has been shown to associate with uncoated endocytotic vesicles via GIPC1, while Myo6(+LI) is recruited to clathrin coated pits/vesicles via Dab2 [6]. Thus, the expression of various Myo6 splice forms in human macrophages further indicates that Myo6 is likely to have multiple functions, including CME, within these cells [7].

In the current study, Myo6 was found to be distributed throughout the cytoplasmic pool in both THP-1 macrophages and HMDM, with particular localisation to actin-generated protrusions of the plasma membrane, and possibly membrane ruffles (Figure 5), often expressing a punctuate pattern in the cell periphery indicative of association with vesicular structures, as noted previously [6, 14, 15]. As expected, Myo6 appeared to be associated with F-actin, particularly at the apical membrane and at putative podosome attachment sites (Figure 6). Similar associations of Myo6 to plasma membrane protrusions and membrane ruffles have been reported in a number of migratory cell types other than macrophages, including epithelial, fibroblast, and ovarian border cells [13, 35], suggesting that it plays an important part in the coordination of the various processes required for cell migration, including cell adhesion and directed cell progression. Chibalina and colleagues [36] have demonstrated that Myo6 functions in endocytosis and vesicle recycling in such cells, and in conjunction with GIPC, it has also been shown to participate in the endocytosis of activated *α*5*β*1 integrin [37], which links it directly to focal adhesion turnover. Buss et al. [13] have also suggested that the association of Myo6 with membrane ruffles in fibroblasts may be indicative of a role in macropinocytosis.

During CME, AP-2 is known to act in the formation of clathrin-coated pits/vesicles [38], while Dab2 can either associate with AP-2 as an accessory protein [39] or act as an adaptor protein in its own right [38]. Dab2 also has numerous functions outside of CME, and so the extent of its association with clathrin-coated vesicles varies between cell types [40, 41]. In this study CLS fluorescence microscopy was used to show that AP-2*α*2 and Dab2 (Figure 4) are located adjacent to the periphery of both THP-1 macrophages and HMDM in a punctate staining pattern, indicative of vesicle association.

To assess the extent of interaction between Myo6, Dab2, and AP-2*α*2 in THP-1 macrophages, double immunofluorescence labeling experiments were used. Dab2 and AP-2*α*2 were shown to be partially colocalised (Figure 7(b)) in a punctate pattern, indicative that Dab2, the direct binding partner of Myo6, is associated with clathrin-coated pits/vesicles in THP-1 macrophages. This finding is consistent with previous work showing that Dab2 is associated with AP-2*α*2 in COS-7 cells [42]. However, although association between Myo6 and AP-1 has been reported in other cell types [7], the present experiments suggest that the two proteins do not have substantially overlapping distributions in macrophages (Figure 7(a)).

Having shown that Myo6, Dab2, AP-2, and GIPC1 are expressed in human macrophages and that there are some interactions between them, we explored the possibility that they might play a part in macrophage foam cell formation. THP-1 macrophages were used for these studies since there are serious technical difficulties in obtaining sufficient HMDM for the types of experiment needed. These cells have some advantages over HMDM in that, because they are genetically homogeneous, there is less variability in their phenotype, and they can be stored indefinitely at −80°C. Another factor which was important for our experiments is that transfection efficiency of THP-1 macrophages with siRNA is much greater than that with HMDM.

The uptake of nLDL and oxLDL by macrophages is known to be mediated by different receptors: nLDL undergoes endocytosis via the LDL receptor (LDLr) which is down-regulated when intracellular cholesterol levels increase, while oxLDL is recognised by unregulated scavenger receptors, thus its uptake is the main driver of foam cell formation [1]. Since numerous studies have demonstrated that nLDL may also directly contribute to foam cell formation [43–45], both nLDL and oxLDL were used in our studies.

The expression of the scavenger receptors CD36, CD68 and scavenger receptor-A (SR-A) has been shown to be upregulated in the presence of oxLDL and to a lesser extent nLDL [46–49], while LDLr expression is downregulated in response to cell exposure by nLDL [50, 51]. In the present work, the effects of nLDL and oxLDL on the expression of mRNA and protein for Myo6, Dab2, AP-2, and GIPC1 in THP-1 macrophages were investigated. Compared to control levels, nLDL significantly decreased mRNA transcript abundance for AP-2*α*2 and GIPC1 (Figures 8(c) and 8(d)), while incubation with oxLDL resulted in reduced levels of Myo6 and AP-2*α*2 mRNA (Figures 8(a) and 8(c)). Little change was seen at the protein level, however, except for a small decrease in Dab2 with nLDL (Figures 8(e)–8(h)). Both Myo6 protein and mRNA levels were significantly lower after incubation with oxLDL compared to nLDL (Figures 8(a) and 8(f)), suggesting that nLDL and oxLDL have different effects on the endocytic machinery, possibly reflecting their uptake via different receptor-mediated endocytic routes [52, 53].

We also examined the effects of knockingdown the expression of Myo6 and associated proteins on the uptake of nLDL and oxLDL. Although substantial knockdown of gene expression was achieved (Figure 9), we did not detect any significant effect on the uptake of lipoprotein type as assessed by fluorescence microscopy (Figure 10). These results, together with our findings of relatively small effects of the lipoproteins on mRNA and protein expression (Figure 8), suggest that Myo6 and its binding partners do not play a major role in the uptake of nLDL or oxLDL by macrophages during induction of foam cell formation.

## 5. Conclusions

The results presented here show that Myo6 and its associated proteins Dab2, AP-2, and GIPC are expressed in human macrophages, displaying similar patterns of expression and subcellular location in both the THP-1 cell line and in primary cells (HMDM). Myo6 was found to be particularly prominent in protrusions of the plasma membrane and membrane ruffles, where it was associated with F-actin. AP-2 and Dab2 appeared to be associated with cell vesicles, and AP-2, but not Myo6, was colocalised with Dab2. Although nLDL and oxLDL had some effects on the expression of the genes for the proteins investigated in macrophages, attenuation of their expression through the action of cognate siRNA molecules did not affect their uptake by the cells. Because the nature of these experiments requires large number of cells, however, HMDM could not be used, THP-1 cells being used instead; for this reason we remain cautious in extrapolating our findings to primary cells, as would be the case with any study involving immortalized cell lines. Nevertheless, the findings presented here on expression and subcellular localisation suggest that, in macrophages, Myo6 functions in cell adhesion and progression as well as in macropinocytosis. Data from the experiments on the uptake of LDL do not support the idea that it plays a major role in foam cell formation.

## Figures and Tables

**Table 1:** Primers, annealing temperatures, and product sizes for PCR. *β*2 mg, *β*2 microglobulin.

Target gene	Forward primer sequence (5′-3′)	Reverse primer sequence (5′-3′)	Annealing temp (°C)		Product size (bp)
			−*β*2 mg	+*β*2 mg	
AP-2*α*2	AGCACAGAAGAACCCAGAAGAG	AGCAGTCTCAGCAGTTTGACAG	60.3	58.5	153
*β*-actin	AGAAAATCTGGCACCACACC	GGGGTGTTGAAGGTCTCAAA	57.3	—	142
*β*2-microglobulin	GTGCTCGCGCTACTCTCTCT	TCAATGTCGGATGGATGAAA	57.0	—	143
Dab2	GCAGACTTCTTCTGGGACTTTG	GTAACTGGCAGGGAAACTTGTC	60.3	58.5	165
GAPDH	AGAACATCATCCCTGCCTCTACT	GATGTCATCATATTTGGCAGGTT	56.5	—	164
GIPC1	GATGACCTGCTGGAGAGTTACA	CCAGACGTCAAAGACGAACTC	60.1	58.5	162
Myo6	GGTTTGGATGATGAAGAAAAGC	CAAACCCAGTAATTCAGCACAA	56.5	56.5	162
Myo6 (small insert)	GCAGCTTGCAGAGAAGAATTT	CTGAGGGTCTTTGTACTGGT	56.6	—	264
Myo6 (large insert)	GTTCTGGAGCAGGAGCGC	AAATTCTTCTCTGCAAGCTGC	58.2	—	312
Ribosomal protein L13a	CCTGGTCTGAGCCCAATAAA	CTTGCTCCCAGCTTCCTATG	58.0	—	144

**Table 2:** Sequences used in siRNA experiments.

siRNA	Target sequence (5′-3′)
Myo6_1	AGAGATAAGTTTATACGGGAA
Myo6_2	AACCGCAAAAGTCCTGAGTAC
Dab2	TGGGAGGTTATGTTTATTTGA
AP-2*α*2	CTCGGATATCCGCAACTGTAA

**Table 3:** Composition of LDL: human LDL was isolated from plasma by sequential density gradient ultracentrifugation, oxidised using CuSO_4_, and labelled with DiI as described in the Methods section. Data shown are the mean ± SEM from 3 preparations.

Parameter	nLDL	oxLDL	DiI-nLDL	DiI-oxLDL
Protein (mg/mL)	2.04 ± 0.01	2.07 ± 0.28	1.09 ± 0.42	1.04 ± 0.28
TC (*μ*mol/mL)	8.37 ± 0.25	7.6 ± 0.51	4.03 ± 1.93	3.87 ± 1.13
TBARS (nmol MDA/mg protein)	0.90 ± 0.25	16.83 ± 3.17**		

**Indicates oxLDL value is significantly different from nLDL value (*P* < 0.01).
